# Can Essential Oils Provide an Alternative Adjuvant Therapy for COVID-19 Infections and Pain Management at the Same Time?

**DOI:** 10.3390/ph15111387

**Published:** 2022-11-10

**Authors:** Roxana Damiescu, David Y. W. Lee, Thomas Efferth

**Affiliations:** 1 Department of Pharmaceutical Biology, Institute of Pharmaceutical and Biomedical Sciences, Johannes Gutenberg University, Staudinger Weg 5, 55128 Mainz, Germany; 2 Bio-Organic and Natural Products Laboratory, McLean Hospital, Harvard Medical School, 115 Mill Street, Belmont, MA 02478, USA

**Keywords:** addiction, aromatherapy, antiviral activity, inflammation, opioid crisis, pain

## Abstract

The active compounds from essential oils have been an important asset in treating different diseases for many centuries. Nowadays, there are various available formulations used as food supplements to stimulate the immune system. In light of the current pandemic and the large amount of fake news circulating the internet, it is important to analyze which of the active compounds from essential oils can be successfully used in the treatment of COVID-19 infections. We analyzed the current literature on the effects of essential oils against the new SARS-CoV-2 virus to gain a better understanding of the underlying mechanisms of these compounds and establish their possible antiviral efficacy. The available studies have highlighted the antiviral potential of active compounds from essential oils, indicating that they could be used as adjuvants in treating various viral infections, including COVID-19, leading to a milder course of the disease, and improving patients’ outcomes. At the same time, these compounds relieve pain and lift the mood in comorbid patients suffering from opioid addiction. Essential oils might be useful as adjuvant tools, not only against SARS-CoV-2 but also for a subset of especially vulnerable patients affected with both COVID-19 and opioid addiction. However, randomized clinical trials are needed to determine their efficacy and develop standardized high-quality preparations that can be safely administered to the general population.

## 1. Introduction

As cases of SARS-CoV-2 infections are still drastically multiplying, despite increasing numbers of vaccinated people, the pressure to find effective medications grows daily. Essential oils from medicinal plants have been used for centuries for their pharmaceutical properties. Therefore, phytochemical compounds have been intensively investigated in the search for alternatives to treat COVID-19. In this context, aromatherapy, which is based on essential oils, has come into the center of interest.

Aromatherapy uses natural essential oils and essences from specific parts of plants such as roots, flowers, leaves, bark, or fruits. Wild or cultivated useful plants can be used. Essential oils are usually complex mixtures of numerous monoterpenes.

Essential oils have been used since time immemorial and in all cultures worldwide. The Egyptians used balsamic oils in medicine, for embalming their dead, and for other ritual religious practices as early as 4500 BC. The Ebers Papyrus (15th century BC) described the antiseptic and antibiotic effects of many essential oils. They were used in China, India, Persia, Greece, and the Roman Empire. Aromatherapy fell into disuse in the European Middle Ages and was revived in the 20th century by the French chemist René Maurice Gattefossé. He described the properties of essential oils, which are still considered the main effects today, i.e., antiseptic, antibacterial, antiviral, and anti-inflammatory effects.

Essential oils are extracted from plants by steam distillation or cold pressing and are usually mixed with fatty oils as carrier oils (e.g., almond, soybean, or wheat germ oil), as essential oils are volatile. In massage, essential oils are absorbed through the skin, where they stimulate nerves and blood circulation. When used for inhalation and facial steam baths, they diffuse not only through the skin but also through internal nasal passages into the tissues. This application has proven effective for colds and could also be considered for COVID-19 treatment. Furthermore, essential oils are used in plant paste poultices, compresses, and as herbal teas and medicinal beverages. Oral ingestion of high concentrations may cause toxic reactions, e.g., dizziness, nausea, vomiting, epileptic fits, allergy, etc. Undesirable contamination (e.g., pesticides, radioactivity, or heavy metals) should be excluded by stringent quality control measures for high-quality preparations [1].

Some essential oils are commercially available as standardized preparations (capsules, gels and creams, and nasal sprays) for treating different viral infections, mostly caused by common cold viruses. Although SARS-CoV-2 attacks the immune system in a far more aggressive fashion than influenza viruses, these medications might still be useful to reduce the severity of the symptoms in combination with other approved drugs. Additionally, essential oils and herbal medicines can be a beneficial alternative or adjuvant therapy in countries with less well developed healthcare systems and limited access to medication, but also for patients with milder courses of the disease, receiving their treatment at home in quarantine. While there are various herbal medicines that are widely used in respiratory infections, there are also a lot of “alternative” treatments circulating on the internet, that lack scientific proof regarding their safety and efficacy and which can produce unwanted side effects in patients. As such, a recent report assessed the benefits/risks of certain herbal medicines traditionally used to improve the symptoms in respiratory infections during the COVID-19 pandemic. The authors selected species listed by recognized organizations (e.g., WHO, EMA) in monographs, but they also included species widely known for their efficacy in ameliorating common cold symptoms (e.g., *Citrus limon* (L.) Burm. f., *Laurus nobilis* L., *Nigella sativa* L., *Mentha x piperita* L.) and compared their efficacy to reference drugs (ibuprofen, paracetamol, and codeine), used to treat COVID-19 symptoms. Based on the available evidence (preclinical and clinical data), the authors evaluated the benefits/risk balance of five herbal medicines as positive (*Althaea officinalis* L., *Commiphora molmol* Engl., *Glycyrrhiza glabra* L., *Hedera helix* L., and *Sambucus nigra* L.) and of 12 other species as promising (*Allium sativum* L., *Andrographis paniculata* Burm. f., *Echinacea angustifolia* DC., *Echinacea purpurea* L., *Eucalyptus globulus* essential oil, *Justicia pectoralis* Jacq., *Magnolia officinalis Rehder & E.H. Wilson*, *Mikania glomerata* Spreng., *Pelargonium sidoides* DC., *Pimpinella anisum* L., *Salix* sp., *Zingiber officinale* Roscoe) [2]. Another study reviewed the chemical composition of herbal medicines known to be active against coronaviruses and evaluated their potential to prevent viral entry and replication as well as inhibit the viral enzymatic system. Many of these compounds have immunomodulatory properties (e.g., phenolics, terpenoid, polysaccharides) and some have been recently used as vaccine adjuvants (polysaccharides, saponins), highlighting the antiviral potential of herbal medicines [3]. Essential oils are known to have a stabilizing effect on mood fluctuations, depression, and anxiety, which has been shown in clinical studies [4,5]. Another important use for essential oils is pain relief [6,7].

It has been demonstrated that the opioid epidemic is a complex public health problem that requires transdisciplinary approaches in order assure successful patient outcomes [8]. The first and hardest step in treating opioid misuse is going through the opioid withdrawal phase. The opioid withdrawal symptoms (OWS) vary in severity, type and duration, but are often severe and lead to the patient’s death, if not treated properly [9]. Some of the symptoms include insomnia, anxiety, dysphoria, fever, sweating, vomiting and diarrhea, muscle spasm and pain, abdominal cramps, tremor, piloerection, rhinorrhea, mydriasis, and tachycardia [9,10]. The treatment of OWS uses mainly μ-opioid receptor full (methadone) and partial (buprenorphine) agonists, α2-adrenergic receptor agonists (clonidine, lofexidine), and ancillary medications for anxiety and sleep (benzodiazepines), muscles cramps (ibuprofen), nausea and vomiting (bismuth subsalicylate, ondansetron) [11]. However, essential oils could be used as both adjuvants for pain management and instead of the ancillary medication to reduce OWS.

While more than 300,000 papers have been published since the outbreak of SARS-CoV-2, the role of aromatherapy as a treatment option has been astonishingly neglected [12,13,14]. The situation is comparable to the application of aromatherapy to reduce opioid requirements [15,16,17,18,19,20,21,22]. Therefore, the intention of this review article is to point out the not fully recognized potential of aromatherapy and essential oils for a specifically vulnerable subset of COVID-19 patients who also suffer from opioid misuse and addiction.

## 2. In Silico Effectiveness of Essential Oil Constituents

Da Silva et al. performed a molecular docking analysis of 171 essential oil components against the following six protein targets: the binding domain of the SARS-CoV-2 spike protein, SARS-CoV-2 endoribonuclease, SARS-CoV-2 main protease, SARS-CoV-2 ADP-ribose-1-phosphatase, SARS-CoV-2 RNA-dependent RNA polymerase, and human angiotensin-converting enzyme. Although, sesquiterpenes (E, E)-α-farnesene, (E)-β-farnesene, and (E, E)-farnesol had the highest affinity towards the targets of interest, the binding affinities were still weak. Therefore, these compounds likely do not inhibit the virus as single compounds. but may instead reveal activity as a compound mixture [23]. Another docking study analyzed the inhibitory effects of various major components from essential oils (star anise, cinnamon, tea tree, lavender, thyme, mint, etc.) against the S1 unit of the spike protein, which is responsible for the binding to the angiotensin converting enzyme 2 (ACE2). Monoterpenes (carvacrol, thymol) and monoterpene alcohol (geraniol), phenylpropanoids (anethole, cinnamaldehyde, cinnamyl acetate), l-4-terpineol, and other terpenes (camphene, menthol, pulegone) prevented viral replication in the host cells [24]. Many of these essential oils are already known for their antiviral activity. The antiviral activity of carvacrol on the SARS-CoV-2 main protease was also confirmed by a second study using molecular docking and molecular dynamics, which also assessed the ADME (absorption, distribution, metabolism, and excretion) properties. Cinnamaldehyde, carvacrol, eugenol and menthol also suppress macrophage recruitment and the production of different proinflammatory cytokines in the bronchoalveolar lavage fluid, having anti-inflammatory and beneficial effects on the lungs [25]. Carvacrol is a compound that has already been analyzed for its antimicrobial potential [26]. Eucalyptus oil is another essential oil, which is worth a second look, as it has already been used in the treatment of various respiratory diseases. Besides its antimicrobial activity, one of its main active components 1,8-cineol (eucalyptol) has myorelaxant, analgesic, anti-inflammatory, and immunomodulatory effects and also reduces monocytes and macrophage recruitment. Inhalation of 1,8-cineol improves the quality of life in patients suffering from asthma and COPD and is recommended as adjunctive therapy. In silico studies have described the binding of 1,8-cineole and jensenone (another active compound from eucalyptus oil) to the SARS-CoV-2 main protease and forming hydrogen bonds, hydrophobic and ionic interactions [25]. A further in silico screening used natural compounds (isothymol, thymol, limonene, p-cymene, and γ-terpinene) from *Ammoides verticillate* Briq. originating from Algeria to inhibit ACE2 in silico. Isothymol showed the most promising results, having good drug-likeness properties and high binding affinity, which were comparable to captopril and chloroquine [27]. The essential oil from *Thymus vulgaris* L. (containing thymol, carvacrol, p-cymene, γ-terpinene, and β-linalool) was tested in vitro for its therapeutical potential in feline infectious peritonitis (FIP) caused by coronavirus. As cases of feline infections with the SARS-CoV-2 have already been reported and treatment options are very limited, it is important to devise therapeutic alternatives to treat not only human but also animal COVID-19 infections [28].

Garlic has been used for centuries, both for its therapeutic effects and as a spice contains various organosulfur compounds with strong immunomodulatory, antioxidant, antibacterial, antiviral, antifungal, hypoglycemic, and hypotensive effects. These effects were confirmed using different garlic preparations (fresh/dried garlic, garlic juice, oil, powder, tincture, garlic oil macerate, and aged garlic extract). Garlic contains more than 30 sulfur compounds, which can be divided into two chemical classes (L-cysteine sulfoxides and γ-glutamyl-L-cysteine peptides) with allicin as the major active compound. Garlic also contains non-sulfur compounds such as lectins, polysaccharides, saponins, flavonoids (quercetin), vitamins, and minerals [29]. Quercetin is available as a food supplement and has already been used in various countries as an adjuvant to treat COVID-19. Molecular docking revealed that 17 organosulfur compounds from garlic (99.4% of garlic essential oil components) can strongly interact and block ACE2 protein and SARS-CoV-2 main protease [30]. ACE2 in the host cells and SARS-CoV-2 main protease represent valuable targets to develop new drug candidates. *Melaleuca cajuputi* Powell is a plant common in southeast Asia. Through GC–MS analyses, 24 main compounds were identified in the essential oil of *M. cajuputi*, including terpineol, cineol, β-selinenol, α-eudesmol, γ-eudesmol, and guaiol. The docking results determined that ACE2 protein and SARS-CoV-2 main protease can be blocked, thereby preventing the viral replication through the synergistic interaction of 10 of these compounds. According to the screening program, terpineol, guaiol and linalool have the strongest inhibitory effect, followed by cineol, β-selinenol, and α- and γ-eudesmol [31].

Recent molecular docking studies indicated further possible anti-SARS-CoV-2 candidates from Brazilian flora, some of which are already documented for their general antiviral properties (e.g., *Baccharis dracunculifolia* DC, *Santolina insularis* (Gennari ex Fiori) Arrigoni, and *Fortunella margarita* Lour.). The best results were obtained for (E)-α-atlantone, α-amorphene, α-cadinene, α-calacorene, α-muurolene, 14-hydroxy-α-muurolene, allo-aromadendrene epoxide, amorpha-4,9-dien-2-ol, aristochene, azulenol, germacrene A, guaia-6,9-diene, hedycaryol, and humulene epoxide II [32]. These results play a key role for the Brazilian population in the fight against COVID-19, since many of the species containing these essential oils are traditionally used. In addition, another promising candidate is Buriti oil extracted from *Mauritia flexuosa* L., a native plant from the Amazon region, which is famous for its biodiversity. Buriti oil is rich in carotene compounds (13-cis-β-carotene, 9-cis-β-carotene, and α-carotene), which interact with the main viral peptidase [33]. One of the world’s smallest countries, the Seychelles islands, offers another possible candidate against COVID-19. *Lodoicea maldivica* (J.F.Gmel.) Pers. (Arecaceae), a palm called ‘Coco de Mer’, contains high amounts of acyclic, bicyclic and monocyclic sesquiterpenoids (β-caryophyllene, bicyclogermacrene) and monoterpenes, known for their antimicrobial properties [34].

## 3. The Pathway from In Silico to In Vivo

Although molecular docking plays an important role in drug discovery, in vitro and in vivo tests and clinical investigations play more decisive roles for the further drug development process. The antiviral activity of organosulfur compounds (OSCs) from garlic has been investigated using different types of cells (VERO, HeLA, Huh-7, and U937) as well as animal studies (of mice), in order to gain a better understanding of the underlying mechanisms. OSCs inhibit the activity of different classes of viruses (including influenza and rhinovirus, corona-, rota-, herpes-, cytomegalo-, hepatitis A virus, and measles). The involved mechanisms include preventing viral entry, fusion and replication in the host cells, blocking viral RNA polymerase and reverse transcriptase, and inducing apoptosis as well as stimulating the host immune response. Additionally, immunomodulatory and anti-inflammatory effects have been induced by activating macrophages and natural killer cells, enhancing the activity of B and T cells OSCs, and activation of anti-inflammatory cytokines [29]. Various clinical trials have assessed the efficacy of garlic extracts in viral diseases such as acute respiratory viral infections, flu, hepatitis B and C and human papillomavirus-induced recalcitrant multiple common warts (RMCW), using different administration routes (oral, nasal, or topical) and doses. The severity of the infections was significantly reduced in the case of common cold and flu infections. In patients suffering from chronic hepatitis, the administration of garlic extract improved the serum levels of aminotransferase (ALT and AST). Furthermore, lipid garlic extract eradicated genital RMCW significantly (70%) better than cryotherapy [29].

Analyses of essential oils against coronaviruses were started after the outbreaks of SARS-CoV in 2003 and MERS-CoV in 2012. HeLa-CEACAM1a (HeLa-epithelial carcinoembryonic antigen-related cell adhesion molecule 1a) cells were infected with MHV-A59 (mouse hepatitis virus-A59) to evaluate the effects of essential oils from *Citrus X sinesis* L., *Anthemis hyalina* DC, and *Nigella sativa* L. on the replication of coronavirus and expression of transient receptor potential protein (*TRP*) genes. The analysis revealed a significant downregulation of *TRP* gene expression and virus load by all three extracts. Furthermore, the virus load was almost undetectable upon treatment with *Anthemis hyaline* extract [35]. *N. sativa* contains several active components such as thymoquinone, nigellone, α-hederin, carvacrol, α- and β-pinene, and thymol, which have anti-inflammatory, antihistaminic, antimicrobial, immunomodulatory and antioxidant effects. Patients with co-morbidities (respiratory and cardiovascular diseases and diabetes) are at a higher risk of developing severe COVID-19 infections, as these cases are often accompanied by bacterial coinfections (pneumonia or sepsis). However, animal experiments have showed beneficial effects of *N. sativa* extracts in reducing inflammation, oxidative stress, and release of histamine, modulating autophagy, improving serum glucose, and cholesterol levels. Therefore, administration of *N. sativa* oil as a supplement may have beneficial effects in patients with comorbidities [36]. Furthermore, in a clinical trial, patients suffering from mild COVID-19 infection received capsules containing 500 mg of *Nigella sativa* oil twice a day for 10 days. Patients receiving *Nigella sativa* oil had reduced COVID-19 symptoms and recovered faster from the infection, compared to the control group [37].

In another investigation, the chemical composition of essential oils of seven Lebanese species (*Laurus nobilis* L., *Juniperus oxycedrus* L., *Thuja orientalis* L., *Cupressus sempervirens* L., *Pistacia palaestina* Boiss., *Salvia officinalis* L., and *Satureja thymbra* L.) was determined by GC–MS analysis, in order to analyze their cytotoxic and antiviral activity against SARS-CoV. *L. nobilis*, which contains β-ocimene, 1,8-cineole, α-pinene, and β-pinene, had a moderate to low antiviral efficiency [38].

*Melaleuca alternifolia* (Maiden and Betch) (tea tree) oil is another candidate for alternative treatments against COVID-19. The compounds (α-terpineol, terpinene-4-ol, and terpinolene) of *M. alternifolia* had efficient in vitro and in vivo efficacy against HSV-1 and HSV-2, and even against acyclovir-resistant strains [39]. Tea tree oil inhibited the replication of influenza virus A/PR/8 subtype H1N1 in vitro by preventing endosome acidification and membrane fusion. It revealed high antiviral efficacy in aerosol and vaporized form, as it inactivated 99% of the influenza A virus. Additionally, the essential oils of *M. angustifolia* inhibited the production of pro-inflammatory cytokines (IL-1β, IL-6, and IL-10) in lipopolysaccharide-stimulated macrophages, demonstrating anti-inflammatory and immunomodulatory effects [40]. The antimicrobial potential of tea tree oil was further highlighted by in situ analyses. Accordingly, tea tree oil films on keyboard surfaces significantly reduced the contamination and transfer of pathogens (*S. aureus*, MRSA, and *Enterobacteriaceae*) to patients treated in an intensive care unit (ICU). Thus, tea tree oil could be used in combination with other surface cleaning products in ICUs to control bacterial spread [41].

As the ACE2 receptor is used by the virus as an entry gate into the host cells, it is important to know whether any compounds exist that could stop this step. The effect of 10 essential oils in blocking ACE2 has been assessed by using human HT-29 colorectal adenocarcinoma cells, which endogenously express ACE2. Although geranium and lemon essential oils had the highest ACE2 inhibitory effect, with low cytotoxic effect at up to 100 µM, other essential oils also showed promising results, including eucalyptus, juniper and tea tree oil, proving that they can block SARS-CoV-2 activity [42].

Many of these essential oils have proven efficiency against viruses, bacteria and fungi and are available in different pharmaceutical formulations (mouthwash/gargle solution or nasal spray). Various clinical trials have evaluated the efficacy of antimicrobial mouthwashes and nasal sprays during this pandemic. Mouth rinses with antiseptic solutions are recommended before a dental procedure because they reduce the amount of microbes in the oral cavity. These solutions often contain essential oils, chlorhexidine, povidone–iodine, or hydrogen peroxide. Mouth rinses containing certain essential oil components (carvacrol, myrtenol, caryophyllene, and pinocarveol) inhibited the S1 unit of the spike protein in silico [43]. Furthermore, in vitro studies have proved the potential of Listerine^®^ (an ethanolic solution containing menthol, thymol, eucalyptol, and methyl salicylate) to significantly reduce SARS-CoV-2 activity, in comparison to the use of ethanol alone. Clinical trials are still ongoing to establish the potential of these compounds in reducing SARS-CoV-2 infections [44]. According to the Cochrane Special Collection, the use of antimicrobial mouthwash and nasal sprays is recommended for healthcare workers who have direct contact with COVID-19 patients, patients undergoing aerosol-generating procedures, and COVID-19-positive patients, whose outcome was improved by these mouthwashes and nasal sprays. Because most of the studies are still ongoing, the results regarding the efficacy of these measures remain to be determined [45].

## 4. Essential Oils in Pain Management and Opioid Dependence

Various essential oils and their active components have already been tested in preclinical and clinical trials (Table 1) for their analgetic role. Carvacrol (5-Isopropyl-2-methylphenol), known for its antimicrobial, analgesic, anti-inflammatory, and antioxidant effects and found in the essential oils of thyme and oregano, is possible candidate. The dose-dependent (50 or 100 mg/kg) antinociceptive effects of carvacrol have been assessed by using different models of pain in mice, such as acetic acid-induced abdominal constriction, formalin, and a hot-plate test. Two studies described a reduction of pain in mice after oral or intraperitoneal administration of carvacrol, but the underlying mechanisms of carvacrol remained unclear, as the acetic acid-induced abdominal constriction and formalin test did not show any effects on the opioid system or the nitric oxide pathway [46,47]. Lower doses of carvacrol (12.5, 25 and 50 mg/kg) administered orally in mice revealed anxiolytic effects without impairing locomotor activity [48]. Animal tests have showed that carvacrol decreased the expression of various inflammatory factors in the brain (IL-1β and TNF-α), regulated brain-derived neurotrophic factor (BDNF), dopamine expression and mitogen-activated protein kinase pathway (MAPK) and activated the nuclear factor E2-related factor (Nrf2), demonstrating its antioxidant and neuroprotective effects [49]. This is an essential role of carvacrol, as the chronic use of opioids leads to neurodegeneration [50,51,52]. Additionally, synthesized GABA esters of thymol and carvacrol have a more potent analgesic effect, compared to the initial monoterpenes. Additionally, these novel esters also revealed a prolonged anticonvulsant activity and, in combination with gidazepam, they have a synergistic effect in preventing seizures [53]. A small clinical trial showed that the analgesic effects of essential oil of *Thymus vulgaris* L. are comparable to ibuprofen in treating dysmenorrhea in young adults [54].

Similar properties have been described for its isomer, thymol (2-isopropyl-5-methylphenol), which also has anti-inflammatory, antioxidant, analgesic, neuroprotective, and anticonvulsant effects [55]. The neuroprotective effects of thymol are partially explained by its potential to reverse the effects of amyloid-β- and rotenone-induced neurotoxicity in rats [56,57], activation of nuclear respiratory factor (Nrf2)/heme oxygenase-1 (HO-1) pathway [58], and interaction with the GABA_A_ receptor, which also explains the anticonvulsant potential [53,59]. Through the above-mentioned effects, plus its antispasmodic and anxiolytic activity, thymol proves to be a promising candidate to reduce the severity of OWS [60].

The analgesic and anti-inflammatory effects of *Nigella sativa* oil and its main component, the opioid receptor agonist thymoquinone [61], have been previously demonstrated. Animal studies using different models of pain (hot-plate, formalin, tail-pinch, and acetic acid-induced writhing tests) or inflammation (carrageenan-induced paw oedema) have confirmed the dose-dependent efficacy of *Nigella sativa* oil and thymoquinone after systemic or local administration [62,63]. Additionally, a small clinical trial reported the successful use of *N. sativa* in treating opioid dependence. Subjects received 500 mg *N. sativa* orally, three times per day, and reported significantly reduced OWS. Additionally, the results also indicated that *N. sativa* can be safely used in the long-term treatment of opioid addiction [64]. However, in order to correctly evaluate the efficacy of *N. sativa* in opioid addiction, further evidence was necessary. Repeated morphine administration stimulated malondialdehyde expression (MDA), nitric oxide (NO) production, and oxidative stress, and reduced intracellular glutathione (GSH) expression and glutathione-peroxidase (GSH-Px) activity. To investigate the effects on morphine-induced tolerance and dependence, mice were administered *N. sativa* oil or thymoquinone and morphine. This co-administration reduced the development of morphine tolerance and dependence and explains the possible mechanisms involved in the brain, as the oil, through thymoquinone, prevents both upregulation of MDA expression, NO overproduction, and oxidative stress, and the reduction of GSH and GSH-PX after repeated morphine administration [65,66]. Similar effects were observed in mice after tramadol and *N. sativa* oil co-administration [67], but this also reduced tramadol-induced hepato- and nephrotoxicity [68]. By using opioid receptor expressing U87 glioblastoma cells, it has been demonstrated that thymoquinone decreased intracellular cAMP concentration after chronic morphine treatment, preventing opioid tolerance and dependence [69]. Repeated tramadol administration induces apoptotic cell death through oxidative stress and cell damage in the brain tissue. The oil prevented tramadol-induced ultrastructural apoptotic changes in the motor cerebral cortex of rats by protecting the cortical neurons and myelinated axons, thereby confirming its therapeutic potential in treating opioid dependence [70]. Furthermore, thymoquinone also prevents epileptic seizures and consequent neurodegeneration through the interaction with GABA receptors [71,72] and it was found to attenuate OWS by clocking the calcium channels [73,74]. A growing body of evidence suggests that *N. sativa* oil and its main component, thymoquinone, are effective and can be safely used, not only during the opioid withdrawal phase but also as therapeutic alternatives in long-term treatment of opioid addiction.

Animals receiving eucalyptus oil (by inhalation or intraperitoneally) were subjected to various pain models to establish its antinociceptive effect. The efficacy of the eucalyptus oil against pain was comparable to morphine, but without affecting motor functions. In addition, by modifying the formalin test, the anti-inflammatory activity could also be determined [20,75]. Advances in nanotechnological drug delivery formulations have improved the therapeutic efficacy of numerous drugs, including essential oils. An optimized nanoemulsion from micellar nanoparticles of eucalyptus oil demonstrated high central and peripheral analgesic activity in rats [76]. In addition, patients who inhaled eucalyptus oil after a total knee replacement surgery described less pain after the surgery and had lower blood pressure [77]. Taken together, eucalyptus essential oil and its main active component, 1,8-cineol, have proved to be promising candidates in pain management.

Morphine-induced conditioned place preference is a method by which to establish the development of morphine tolerance and dependence. This method has been also used to determine the analgesic effects of essential oils. In one study, mice were administered essential oil of *Pimpinella anisum* L. intraperitoneally (0.125–0.5 mL/kg), while in other studies *Zingiber officinale* Roscoe (50 and 100 mg/kg) was administered to rats before they were injected with morphine. The degree of morphine tolerance and dependence was reduced by co-administration of these essential oils [78,79,80], and a synergistic effect of co-administration of ginger extract with morphine has been established [81]. Additionally, ginger extract also reduced the expression levels of proteins involved in neuroinflammation (glial fibrillary acidic protein and p38 mitogen-activated protein kinase) in the nucleus accumbens, caused by chronic morphine administration [82]. Patients with moderate to severe knee pain, who were administered ginger and orange essential oils topically (through massage therapy), reported only short-term pain relief [83]. However, patients with knee osteoarthritis, who received standardized ginger (25 mg) and echinacea (5 mg) supplements for 30 days against inflammation and chronic pain, reported an improvement of pain and quality of life as well as a reduction in knee circumference [84].

Lavender oil is often used in pain management as a non-pharmacological alternative. However, its efficacy depends on the type of pain. Patients suffering from chronic back or neck pain showed a better range of motion and reduced pain after receiving acupressure massage with lavender oil. Lavender aromatherapy effectively reduced pain intensity in women after cesarean section and episiotomy, but also in children after blood draw or tonsillectomy [6]. Additionally, the use of lavender oil in obese patients after laparoscopic adjustable gastric banding reduced their post-operative opioid demand [15]. However, lavender oil showed no significant effect in reducing pain after open-heart surgery or spinal procedures, but it did reduce patients’ anxiety levels [6,17,85]. An animal model of inflammatory and neuropathic pain provides new information on the underlying mechanisms of action of lavender oil in pain management. The results indicate that inhalation of lavender oil reduces hyperalgesia and inflammation by activating the opioid receptors and cannabinoid receptor 2 [21]. Taken together, these data indicate the benefits of aromatherapy combined with other nonpharmacological and/or pharmacological treatments in pain management.

## 5. Conclusions and Future Perspectives

Many of the active compounds from essential oils and plant extracts are available as food supplements to help stimulate the immune system (*Zingiber officinale* Roscoe, *Echinacea angustifolia* DC., *Echinacea purpurea* L., *Allium sativum* L.) and are traditionally used as adjuvant therapy against common cold or flu symptoms (Eucalyptus oil, thyme oil, tea tree oil) and treating muscle soreness and pain (eucalyptus, ginger, thyme oil). Essential oils such as *Thymus vulgaris* L. (thymol, carvacrol p-cymene, γ-terpinene, β-linalool), *Allium sativum* L. (allicin, quercetin), *Melaleuca alternifolia* (Maiden and Betch) (α-terpineol, terpinene-4-ol, terpinolene) and *Nigella sativa* L. (thymoquinone, nigellone, α-hederin, carvacrol, α- and β-pinene, thymol) also show promising results against COVID-19. Furthermore, essential oils from *Thymus vulgaris* L. (carvacrol, thymol), *Zingiber officinalis* Roscoe, *Echinacea purpurea* L., *Eucalyptus globus* Labill. (1,8-cineol) have demonstrated analgesic effects in both preclinical and clinical trials (Table 1), making them perfect candidates to be used as adjuvants in pain management. *Nigella sativa* and its main compound thymoquinone also have a proven potential in long-term treatment of opioid dependence. These results indicate that *N. sativa* oil and *T. vulgaris* oil could be safely used in patients with mild COVID-19 symptoms and those who are suffering from opioid dependence.

The main advantage of these compounds is that they are generally considered safe, and by administering them in combination with other drugs, the risk of viral resistance and tolerance may be minimized. Although essential oils contain many active compounds with different beneficial properties, their use is limited due to various factors including poor solubility, cytotoxicity to normal cells, and lack of standardized dosage and concentrations of the active compounds. Furthermore, their potential side effects such as burning or irritation should not be overlooked. However, the development of essential oil nanoemulsions is promising, as advanced drug delivery devices increase the therapeutic efficacy and allow the production of preparations with standardized dosages and the reduction of side effects [86].

In order to obtain more data regarding the efficacy of essential oils in clinical trials, they must be further tested at standardized doses. There are various countries where products containing essential oils are available on the market as medicines and not only food supplements. This is an important aspect, because the legal distribution as medicines is stricter, but the advantage is that the packages contain much more detailed information about the concentration, dosage, side effects, and possible contraindications, compared to food supplements.

Available and novel formulations could be used as combination therapy against both COVID-19 and acute and chronic pain in patients suffering from opioid addiction. As such, further clinical trials are needed, not only to determine the effective concentration of these compounds but also to assure a the quality and safety of these formulations.

The scientific literature provides a rational basis for aromatherapy and essential oils to be used in patients suffering from both COVID-19 and opioid addiction at the same time. This offers unique opportunities to develop novel strategies for prevention and treatment. In the USA, where the opioid crisis is most pressing, more than half of all patients with depression (including opioid-addicted persons) use complementary and alternative medicine as adjuvant therapy [87,88]. Hence, it is reasonable to assume that their readiness to use aromatherapy is high. Currently, there are multiple dietary supplements available on the market that promise to boost the immune system or relieve pain. While, for some of them, scientific data are available to support these claims, many others lack scientific evidence. As more and more people are turning to alternative medicine as adjuvant therapies, it is necessary to ensure that they have access only to medicines that are efficient in alleviating their discomfort and available in adequate formulations to assure the therapeutic efficacy. This is a reason why essential oils that have already been investigated in preclinical and small clinical trials should be further analyzed in combination with approved pharmacological therapies, to establish their efficacy. Hand-held, portable inhalators, initially developed for asthma and other related symptoms, could be easily used to release essential oils into the nose. While some clinical trials support the effectiveness of essential oils and aromatherapy, others do not [89,90]. Thus, well-designed randomized clinical trials must be performed to prove their efficacy. The combination of aromatherapy with modern devices utilizing pharmaceutical technology has the potential to ease the disease burden described here.

## Figures and Tables

**Table 1:** Essential oil tested in preclinical and clinical trials.

Essential Oils	Active Compounds	Preclinical Trials	Clinical Trials
*Nigella sativa* L.	thymoquinone, nigellone, α-hederin, carvacrol, α- and β-pinene, thymol	In vitro tests on HeLa cells infected with MHV-A59 Animal studies using different pain models	COVID-19 patients with mild symptoms Opioid dependence
*Thymus vulgaris* L.	thymol, carvacrol p-cymene, γ-terpinene, β-linalool	Animal studies using different pain models	Patients suffering from dysmenorrhea
*Eucalyptus globus* Labill.	1,8-cineol, α-pinene, jensenone	In vitro test against SARS-CoV-2 and herpes simplex viruses Animal studies using different pain models	Patients suffering from COPD Patients after a knee replacement
*Zingiber officinale* Roscoe	Gingerol, shogaols, paradols, zingerone	Animal studies verifying morphine tolerance and dependence	Patients with osteoarthritis
*Melaleuca alternifolia*	α-terpineol, terpinene-4-ol, terpinolene	In vitro tests against HSV-1, HSV-2, influenza viruses, SARS-CoV-2	
*Lavandula angustifolia* Mill.	linalool, linalyl acetate, 1,8-cineole, β -ocimene, 88 terpinen-4-ol, and camphor	Animal models of chronic pain	Women after cesarean section Children after blood draw or tonsillectomy Patients after laparoscopic adjustable gastric banding

## Data Availability

Data sharing not applicable.
